# TANC1 methylation as a novel biomarker for the diagnosis of patients with anti-tuberculosis drug-induced liver injury

**DOI:** 10.1038/s41598-021-96869-5

**Published:** 2021-08-31

**Authors:** Dongxue Wu, Yuhong Li, Qi Ren, Shengfei Pei, Lin Wang, Luming Yang, Yingzhi Chong, Shufeng Sun, Jinqi Hao, Fumin Feng

**Affiliations:** 1 School of Public Health, North China University of Science and Technology, Tangshan, 063210 China; 2 College of Nursing and Rehabilitation, North China University of Science and Technology, Tangshan, 063210 China; 3 School of Public Health, Baotou Medical College, Inner Mongolia University of Science and Technology, Baotou City, 014040 Inner Mongolia China; 4 School of Life Science, North China University of Science and Technology, Tangshan, 063210 China

**Keywords:** Epigenetics, Epigenetics, Genetic markers, Genomics, Sequencing, Diagnostic markers, Hepatology

## Abstract

We aimed to elucidate the differences in genomic methylation patterns between ADLI and non-ADLI patients to identify DNA methylation-based biomarkers. Genome-wide DNA methylation patterns were obtained using Infinium MethylationEPIC (EPIC) BeadChip array to analyze 14 peripheral blood samples (7 ADLI cases, 7 non-ADLI controls). Changes in the mRNA and DNA methylation in the target genes of another 120 peripheral blood samples (60 ADLI cases, 60 non-ADLI controls) were analyzed by real-time polymerase chain reaction and pyrosequencing, respectively. A total of 308 hypermethylated CpG sites and 498 hypomethylated CpG sites were identified. Significantly, hypermethylated CpG sites cg06961147 and cg24666046 in TANC1 associated with ADLI was identified by genome-wide DNA methylation profiling. The mRNA expression of TANC1 was lower in the cases compared to the controls. Pyrosequencing validated these two differentially methylated loci, which was consistent with the results from the EPIC BeadChip array. Receiver operating characteristic analysis indicated that the area under the curve of TANC1 (cg06961147, cg24666046, and their combinations) was 0.812, 0.842, and 0.857, respectively. These results indicate that patients with ADLI have different genomic methylation patterns than patients without ADLI. The hypermethylated differentially methylated site cg06961147 combined with cg24666046 in TANC1 provides evidence for the diagnosis of ADLI.

## Introduction

Tuberculosis (TB) remains one of the top 10 causes of death worldwide^1^. Directly observed treatment short-course chemotherapy (DOTS), which emphasizes the use of a combination of isoniazid (H), rifampicin (R), pyrazinamide (Z), and ethambutol (E) for 6 to 8 months, was the most effective strategy^2^. The combination of these medications was most likely to result in an increase in the incidence of ADLI^3^. The incidence of ADLI currently ranges from 2 to 28%, depending on the definition of liver toxicity and the population being studied^4,5^. ADLI has significant side effects, such as interruption of treatment, prolonged disease progression, and development of drug resistance, and may even lead to death^6^. There are currently no sensitive and specific biomarkers for the diagnosis of ADLI, and the diagnosis of ADLI still depends on serum biochemical tests. This is because the identification novel potential non-invasive biomarker for the early diagnosis of ADLI in China, as well as across the world, remains a challenge.

DNA methylation is one of the most stable epigenetic modifications in mammalian cells. It controls a variety of cellular and developmental processes, including embryonic development, X-inactivation, chromosome stability, and imprinting^7^. Over the last decade, aberrant DNA methylation has been shown to be a candidate biomarker of cancer and occurs very early in cancer development^8^. Moreover, it has become clear that DNA methylation is reversible and dynamic as a result of enzymatic DNA de-methylation^9^; therefore, aberrant DNA methylation modifications have attracted increased interest as potential drug targets^10^.

Numerous studies have demonstrated that the hypermethylation of CpG islands in the promoter regions of CYP2E1, CYP2D6, and GSTP1 is associated with the occurrence of ADLI^11,12^. However, these studies have only focused on single or multiple genes, and the research conclusions drawn remain limited. At present, there have been studies using Agilent Human DNA Methylation Microarray 1 × 244 K array for genome-wide DNA methylation. However, the Agilent Human DNA Methylation Microarray 1 × 244 K array focuses only on CGI and differentially methylated regions. It only covers 27,627 human CpG islands and 5081 UMR regions, and cannot reach the resolution of a single base. The EPIC BeadChip not only contains over 850,000 probes and covers the entire CpG islands, promoter, coding region, open chromatin, and enhancer, but also includes CpG sites outside the CpG islands, known differentially methylated region sites, and microRNA promoter regions. Moreover, it has the advantage of single base resolution, which can directly detect the exact site of methylation. It is currently the most suitable DNA methylation research technology for apparent genome-wide association analysis (EWAS) research. At present, there is no comprehensive and systematic genome-wide DNA methylation analysis using EPIC BeadChip array in peripheral blood of ADLI patients As such, in the present study, we chose to use EPIC BeadChip to characterize genome-wide DNA methylation profiles in the peripheral blood of ADLI and non-ADLI patients. The differentially methylated CpG sites (dmCpGs) were identified by differential methylation analysis, and pyrophosphate sequencing was used to verify the selected gene sites. In summary, this study aimed to identify epigenetic changes in peripheral blood samples in order to identify potential biomarkers of ADLI.

## Materials and methods

### Study population and ethics

Patients with newly diagnosed TB over 18 years old hospitalized in the Tangshan Fourth Hospital between March 2016 and July 2017 were recruited. TB was diagnosed based on previously described guidelines^13^. All patients received standardized daily treatment with isoniazid (H), rifampicin (R), pyrazinamide (Z), and ethambutol (E) in the first two months, and H and R on a daily basis in the following four months^14^. The follow-up period for patients was from the beginning of treatment until 6 months later. During this period, patient compliance, the choice of treatment options, tuberculosis-related symptoms, and adverse drug reactions were strictly monitored by trained staff. Liver enzymes and bilirubin, as biomarkers of liver function, were monitored. To this end, 10 mL Peripheral blood samples were collected every two weeks after starting antituberculosis treatment for the initial 2 months and every 4 weeks for the next 4 months, or any time when symptoms and signs of hepatitis developed during treatment^15^. We collected peripheral blood samples from patients at 8–9 am and asked patients to fast for 8–12 h before collecting blood samples to ensure the accuracy of the test results. Detailed demographic and clinical characteristics of the patients were obtained from electronic medical records.

We selected 67 patients, who developed ADLI within 2 to 8 weeks after receiving anti-TB treatment as the liver injury group, and then identified 67 patients without ADLI based on similar characteristics of age, sex, and admission time during the same period^15^. A total of 134 TB patients met the inclusion and exclusion criteria and were included in the study. We first performed EPIC BeadChip array of 7 ADLI patients and 7 age- and sex-matched non-ADLI patients. Subsequently, an independent cohort of 60 patients with ADLI and 60 patients with non-ADLI was used for pyrosequencing. This study was reviewed and approved by the Ethics Committee of North China University of Science and Technology (process no. 14-016). Informed consent was obtained from patients prior to any experimental procedures.

### Inclusion/exclusion criteria

The inclusion criteria were as follows: (1) over 18 years old; (2) newly diagnosed with TB; (3) normal liver function before anti-tuberculosis treatment. The exclusion criteria were as follows: (1) patients with acute hepatitis, cirrhosis of the liver, encephalopathy, or cancer; (2) patients taking concomitant hepatotoxic medications; (3) heavy alcohol intake; (4) patients with severe cardiovascular, cerebrovascular, renal, or thyroid disease.

### Diagnosis of anti-tuberculosis drug-induced liver injury

Hepatotoxicity due to anti-TB drug treatment was not only based on the liver enzyme results, according to the criteria of the American Thoracic Society (ATS)^16^, but also took into account the diagnostic criteria developed by the Centre for Drug Re-evaluation (CDR) of the Chinese State Food and Drug Administration, as well as various previously definitions^2,17^. Specifically, ADLI cases need to meet one of the following criteria: (1) an increase in serum alanine aminotransferase (ALT) or aspartate aminotransferase (AST) that is over threefold^2^ the upper limit of normal (ULN) in the presence of liver injury symptoms; (2) an increase in total bilirubin (TBIL) that is over twofold the ULN in the presence of liver injury symptoms; 3) a fivefold increase in the ULN of serum ALT, AST, or TBIL with or without liver injury symptoms.

### Illumina Infinium MethylationEPIC BeadChip array

Microarray-based DNA methylation profiling was performed using the Illumina Infinium MethylationEPIC (EPIC) BeadChip (Illumina, Inc., San Diego, CA, USA) on 7 paired blood samples. Genomic DNA from peripheral blood samples was extracted using a DNeasy Blood and Tissue Kit (Qiagen, Hilden, Germany). Bisulfite conversion of isolated genomic DNA (500 μg) was performed using the EZ DNA methylation Gold Kit (Zymo Research, Irvine, USA). Bisulfite-converted DNA was then whole-genome amplified, enzymatically fragmented, and hybridized to the array as per the EPIC BeadChip protocol^18^. Subsequent scanning of chips was performed using an Illumina HiScan2000. The raw intensity of the data was determined using GenomeStudio methylation module version 1.9.0 (Illumina, Inc.).

### Methylation EPIC BeadChip data pre-processing

The raw intensity data (IDAT) were imported into R version 3.4.2 and processed using the R/Bioconductor package minfi (version 1.22.1)^19^. Low-quality data (probes with detection P-value > 0.05), probes from the X and Y chromosome, and probes overlapped with single-nucleotide polymorphisms were removed^20^. The background data were normalized using the Noob method^21^ to generate methylation beta (β) values, which were used for subsequent analysis.

### Differential DNA methylation analyses

The β-values were used as an indicator of the methylation of each locus in each sample. Delta beta (Δβ) is defined as the difference in the β values between the two groups, in which the absolute value is directly proportional to the degree of difference. We calculated the mean detection P-value to check the overall data quality. In the present study, dmCpGs between the groups were identified with P < 0.05 and |Δβ|> 0.10. Subsequently, dmCpGs located in the promoter regions (promoters were defined as regions located between 1500 bp upstream of TSS and 500 bp downstream of transcriptional start sites (TSS)) and genes containing multiple differentially methylated probes were selected as our candidate CpG sites.

### Real-time quantitative polymerase chain reaction

RNA was extracted using TRIzol reagent (Invitrogen, Grand Island, NY, USA). cDNA was generated using the PrimerScript RT Kit with the gDNA Eraser (DRR047A, Takara, Dalian, China). Subsequently, the cDNA was amplified using SYBR Primix EX Taq II (RR820A; Takara, Dalian, China). The data were normalized to the reference gene glyceraldehyde 3-phosphate dehydrogenase (GAPDH). The primers are shown in Supporting Information Table S2. The 2^−∆∆Ct^^22^ method was used to calculate the relative level of target gene expression.

### DNA pyrosequencing

DNA samples were bisulfite converted using the Zymo EZ DNA Methylation Kit (Zymo Research) as mentioned previously. PCR reaction (25 μL) was performed using TaKaRa EpiTaq HS (for bisulfite-treated DNA) (R110A; Takara, Dalian, China), according to the manufacturer’s instructions. The primer sequences and reaction conditions for all pyrosequencing assays are listed in Supporting Information Table S3. Biotin-labeled PCR products were captured using Streptavidin Sepharose beads (GE Healthcare Life Sciences, Pittsburgh, PA, USA), and single-strand DNA (ssDNA) was made with a Pyrosequencing Vacuum Prep Tool (Qiagen, Hilden, Germany). Sequencing primers were subsequently annealed to the ssDNA template and pyrosequenced using the PyroMark Q96 system (Qiagen, Hilden, Germany). All pyrosequencing data were analyzed using CpG run-analysis on PyroMark Q96 2.5.8 software.

### Ingenuity pathway analysis

Ingenuity pathway analysis (IPA) software (QIAGEN Inc. software version 65367011, https://www.qiagenbioinformatics.com/products/ingenuity-pathway-analysis) was used to analyze significant differentially methylated genes to elucidate the canonical pathways, upstream regulators, disease and biological functions, and networks. The algorithms developed for use in IPA have been described by Kramer et al.^23^.

### Statistical analysis^15^

SPSS software version 22.0 (IBM Corp, Armonk, NY, USA) was used for data analysis. Continuous variables were described as the mean ± standard deviation (SD) or as the median with interquartile range (IQR). Differences between groups were analyzed using the paired-samples t-test or Wilcoxon signed-rank test. Categorized variables are expressed as numbers and percentages and were analyzed using the chi-squared (*χ*^2^) test or Fisher’s exact test. Receiver operating characteristic (ROC) curve analysis was used to determine the AUC, sensitivity, and specificity of peripheral blood DNA methylation. Logistic regression with forward stepwise analysis was used to further establish the diagnostic panel. A two-tailed P-value < 0.05 was considered statistically significant.

### Ethics approval and consent to participate

Approval was obtained from the ethics committee of the North China University of Science and Technology. The procedures used in this study adhere to the tenets of the Declaration of Helsinki. Informed consent was obtained from all individual participants included in the study.

## Results

### Characteristics of the study population

The characteristics of the two study populations used in EPIC BeadChip for discovery, pyrosequencing, and validation are presented in Table 1. All participants were native Han Chinese. In the discovery and validation groups, there were no significant differences between the cases and controls in terms of smoking status, drinking status, ALP levels, TBIL levels, albumin (ALB) levels, or TP levels. However, statistically significant differences in the levels of ALT and AST were found when the cases were compared to controls.

**Table 1:** Clinical and biochemical characteristics of patients.

	EPIC array for discovery group			Pyrosequencing for validation group		
	Cases group (n = 7)	Control group (n = 7)	*P*	Cases group (n = 60)	Control group (n = 60)	*P*
**Clinical characteristics**						
Age (year) M (IQR)	42.0 (30.0–55.0)	44.0 (28.0–52.0)	N/A	41.5 (25.3–55.8)	43.5 (26.3–56.5)	0.787
**Gender, n (%)**						
Male	4 (57.1)	4 (57.1)	N/A	41 (68.3)	41 (68.3)	1.000
Female	3 (42.9)	3 (42.9)		19 (31.7)	19 (31.7)	
**Smoking, n (%)**						
Yes	1 (14.3)	1 (14.3)	N/A	38 (80.0)	35 (75.0)	0.575
NO	6 (85.7)	6 (85.7)		22 (20.0)	25 (25.0)	
**Drinking, n (%)**						
Yes	1 (14.3)	2 (28.6)	N/A	34 (73.3)	33 (71.7)	0.854
No	6 (85.7)	5 (71.4)		26 (26.7)	27 (28.2)	
BMI (Kg/m^2^) M (IQR)	20.8 (19.1–22.1)	20.3 (17.7–21.0)	0.735	20.1 (18.9–21.9)	20.4 (18.3–21.5)	0.800
**Biochemical characteristics**						
ALT (U/L) M (IQR)	255.0 (123.0–397.0)	13.0 (8.0–15.0)	0.018	161.0 (125.3–224.0)	11.5 (8.0–18.8)	< 0.001
AST (U/L) M (IQR)	153.0 (89.0–502.0)	16.0 (16.0–19.0)	0.018	106.2 (67.3–153.7)	16.5 (13.0–21.8)	< 0.001
ALP (U/L) M (IQR))	79.0 (43.9–131.0)	80.0 (75.6–94.0)	0.735	78.0 (60.0–96.3)	81.0 (71.0–97.5)	0.373
TBIL (μmol/L) M (IQR)	8.9 (6.8–16.4)	9.1 (5.1–12.8)	0.866	11.0 (6.8–18.8)	9.4 (6.0–12.6)	0.051
ALB (g/L) M (IQR)	42.0 (40.6–44.4)	38.1 (37.0–42.0)	0.091	41.6 (37.6–43.6)	41.5 (37.2–44.2)	0.702
TP (g/L) M (IQR))	68.0 (65.7–76.3)	72.0 (60.1–79.8)	0.866	68.0 (61.6–71.9)	69.1 (65.6–76.2)	0.139

### Differential DNA methylation patterns between ADLI cases and controls

**Figure 1:**
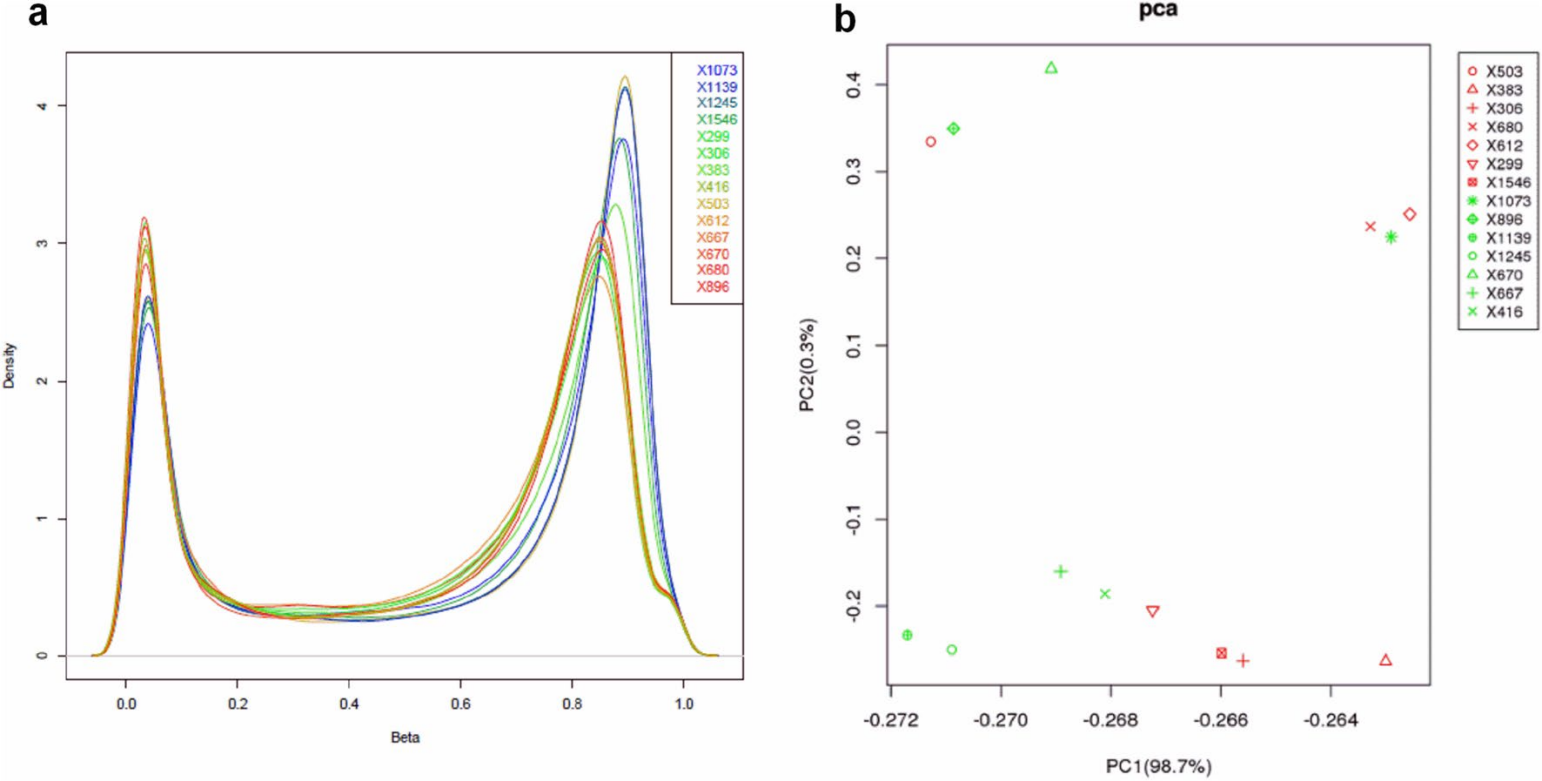
Statistical methylation patterns of cases and controls. (**a**) Density plot of β-values for all probes in the EPIC arrays for all 14 individuals. (**b**) PCA of whole DNA methylomes allows for the differentiation between ADLI cases (N = 7, red shapes) and matched controls (N = 7, green shapes).

To clarify the difference in the levels of methylation between the ADLI patients and the control group, we analyzed the methylation status of 866,091 CpG sites in 7 paired blood samples from 14 subjects using the EPIC BeadChip array. The high quality of the samples is reflected in the pattern and highly comparable distribution shown by the density plot of the β-value of the probe (Fig. 1a). Principal component analysis (PCA) of the full methylomes clearly differentiated between ADLI patients and the controls, as shown in Fig. 1b. Subsequent to data pre-processing and quality filtering, a final data matrix comprised β-values (methylation levels) across 841,456 loci in 14 blood samples was created for further statistical analyses.

**Figure 2:**
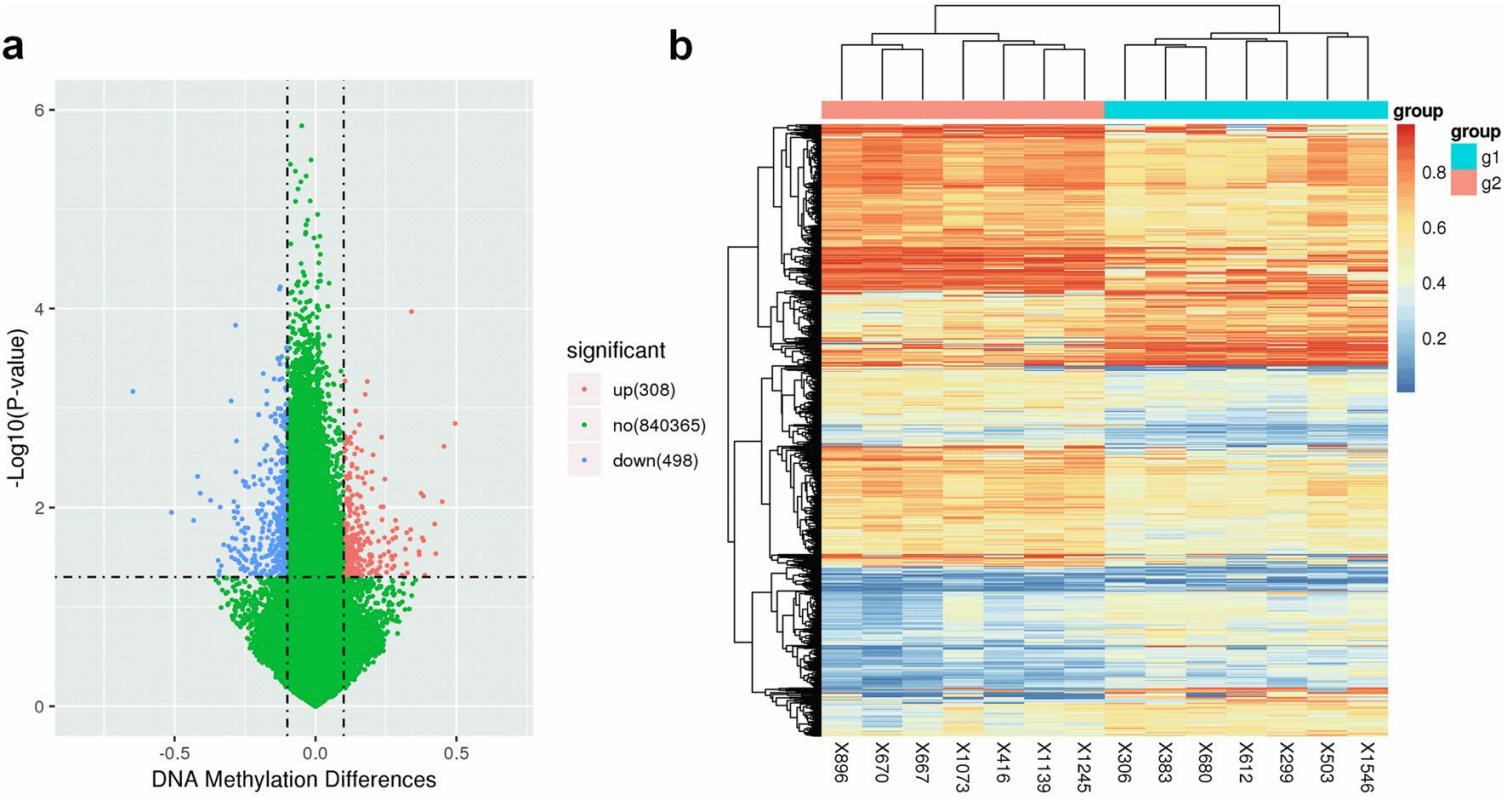
Visualization of differentially methylated probes. (**a**) Volcano plot showing differentially methylated CpG sites. The x-axis represents the magnitude of the difference in signal intensity between the groups for each probe in the microarray, expressed as: ∆β = β (cases) − β (controls). The y-axis represents the − log10 (P‑value), with a P‑value of 0.05. Significant difference sites (*P* < 0.05 and |Δβ|> 0.10) are highlighted in red and blue. Sites highlighted in red and blue are those that were hypermethylated and hypomethylated compared to the controls, respectively. (**b**) Unsupervised hierarchical clustering of the 806 most variable CpG sites derived from samples distinguishes ADLI (N = 7) cases and matched controls (N = 7). Two different groups are represented: group 1 (X1546, X299, X306, X680, X383, X612, and X503), i.e. the case group (columns marked in red), and group 2 (X1245, X416, X1139, X670, X667, X896, and X1073), i.e. the control group (columns marked in blue). Each column represents one sample (sample names below), and each horizontal line represents the methylation levels of a given CpG locus across samples. Methylation levels are expressed as β-values from 0 to 1 (blue and red, un-methylated, and completely methylated, respectively).

Next, a pooled t-test was used to identify the differentially methylated CpG loci between the ADLI patients and the matched control blood samples, resulting in the identification of 806 significantly differentially methylated CpG loci (*P* < 0.05 and |Δβ|> 0.10) (Fig. 2a). Among the 806 dmCpGs, 308 were hypermethylated and 498 were hypomethylated (Fig. 2a). Furthermore, unsupervised hierarchical clustering of the 806 dmCpGs showed a clear segregation between ADLI and without ADLI (Fig. 2b), confirming that the DNA methylation patterns of leukocytes in ADLI patients differ from those of non-ADLI patients.

**Figure 3:**
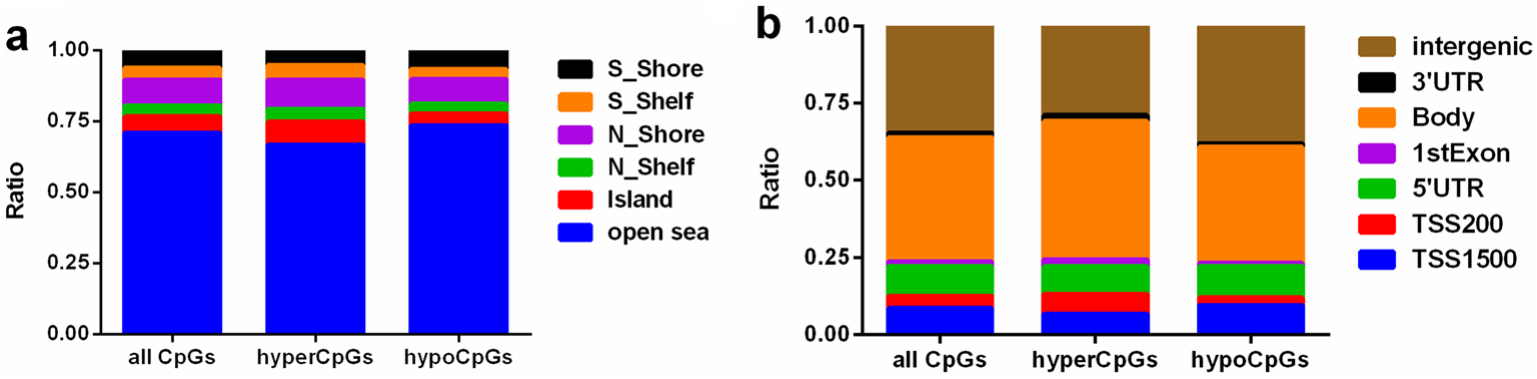
Stacked bar plots depicting the percentage of differentially methylated CpG sites according to their CpG island-related or gene location-related status. **(a)** Percentage of dmCpGs according to their location in relation to CpG islands. (**b**) Percentage of dmCpGs according to genic location.

Figure 3a depicts the genomic distribution of dmCpGs and distinguishes between CpG island-related regions and gene-related regions. Both hypomethylated and hypermethylated dmCpGs were found to be enriched in open sea regions rather than the CpG islands (Fig. 3a). In terms of gene-related locations, hypermethylated and hypomethylated CpGs were preferentially situated in gene body regions and intergenic regions, respectively, and both were impoverished at the 3’UTR regions (Fig. 3b).

We found a total of 53 genes, including multiple differentially methylated CpGs. (see Supporting Information Table S1). These genes were located in different gene-related regions. We focused on the analysis of dmCpGs in the promoter since many studies have suggested that promoter methylation significantly affects the levels of gene expression. A total of 10 genes contained two or more dmCpGs in the promoter regions, including 5 hypomethylated genes (C22orf39, HCG27, PKD1L2, BIRC7, and LOC100507140) and 5 hypermethylated genes (C1orf141, CD177, FMOD, LOC102723376, and TANC1) (Table 2).

**Table 2:** Candidate differentially methylated sites identified between cases and controls.

CpG Locus	Refseq Gene	Refseq Accession	CHR Position	Refseq Group	CpG Island Region	Δβ
cg01641381	C1orf141	NM_001276352	chr1:67601159	TSS1500	S_Shore	0.106157
cg09433222	C1orf141	NM_001276352	chr1:67600822	TSS200	S_Shore	0.104622
cg17623879	C22orf39	NM_001166242	chr22:19436004	TSS1500	S_Shore	− 0.10521
cg26643617	C22orf39	NM_173793	chr22:19436224	TSS1500	S_Shore	− 0.11274
cg15742245	CD177	NM_020406	chr19:43857717	TSS200	N/A	0.105573
cg22537604	CD177	NM_020406	chr19:43857074	TSS1500	N/A	0.101561
cg16289210	FMOD	NM_002023	chr1:203320396	TSS200	N/A	0.163329
cg22705746	FMOD	NM_002023	chr1:203320506	TSS1500	N/A	0.164446
cg26987645	FMOD	NM_002023	Chr1:203320386	TSS200	N/A	0.200169
cg27387030	FMOD	NM_002023	chr1:203320541	TSS1500	N/A	0.173274
cg01739509	FMOD	NM_002023	chr1:203320732	TSS200	N/A	0.128983
cg03062822	FMOD	NM_002023	chr1:203320651	TSS200	N/A	0.149878
cg11897689	FMOD	NM_002023	chr1:203320661	TSS200	N/A	0.120835
cg21089380	FMOD	NM_002023	chr1:203320659	TSS200	N/A	0.129496
cg03030317	HCG27	NR_026791	chr6:31164924	TSS1500	N_Shore	− 0.10403
cg12771717	HCG27	NR_026791	chr6:31164947	TSS1500	N_Shore	− 0.11184
cg15244183	LOC102723376	NR_110795	chr18:11143	TSS1500	N_Shore	0.200666
cg21218768	LOC102723376	NR_110795	chr18:11155	TSS1500	N_Shore	0.114235
cg07091758	PKD1L2	NM_052892	chr16:81254209	TSS1500	N/A	− 0.12216
cg14891093	PKD1L2	NM_001076780	chr16:81254031	TSS200	N/A	− 0.10298
cg20109624	PKD1L2	NM_001076780	chr16:81254265	TSS1500	N/A	− 0.15096
cg21044139	PKD1L2	NM_001076780	chr16:81254139	TSS200	N/A	− 0.13771
cg18472223	LOC100507140	NR_037886	chr2:201600643	TSS1500	N/A	− 0.21112
cg20517941	LOC100507140	NR_037886	chr2:201600636	TSS1500	N/A	− 0.21025
cg06961147	TANC1	NM_001145909	chr2:159824263	TSS1500	N_Shore	0.104792
cg24666046	TANC1	NM_001145909	chr2:159823997	TSS1500	N_Shore	0.234293

Δβ = mean β value (case) − mean β value (control). TSS, transcription start site.

### Expression levels of candidate genes

**Figure 4:**
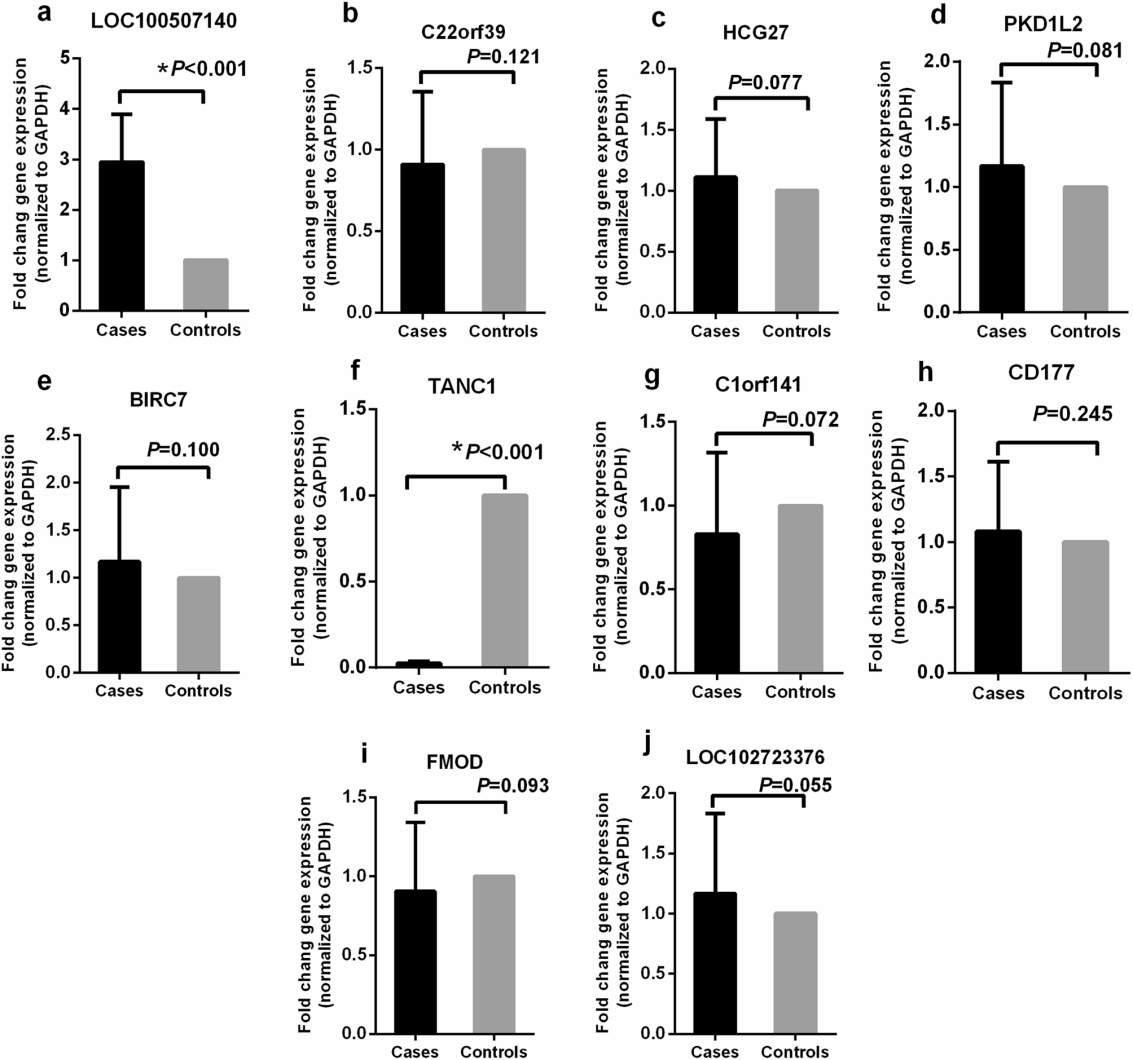
Expression of selected genes in the blood samples of cases and controls. Relative mRNA quantification of 10 candidate genes using RT-qPCR in cases (N = 60) compared to controls (N = 60) blood samples. Data are represented as mean ± SD. *P < 0.001.

To determine whether the methylation of a candidate gene affected the gene expression levels, the mRNA expression of these target genes was analyzed using RT-PCR. For one of the hypomethylated differentially methylated genes, LOC100507140, it expression was higher in the ADLI patients compared to the controls (Fig. 4a), while the expression of C22orf39, HCG27, PKD1L2, and BIRC7 showed no significant difference (Fig. 4b–e). Among the hypermethylated differentially methylated genes (DMGs), only TANC1 expression was low in the ADLI patients compared to controls (Fig. 4 f), while the expression levels of the other four genes were not significantly different (Fig. 4g–j). As such, we selected LOC100507140 (2 sites) and TANC1 (2 sites) for the subsequent pyrosequencing experiments.

### Pyrosequencing validation of differentially methylated ADLI-associated sites

**Figure 5:**
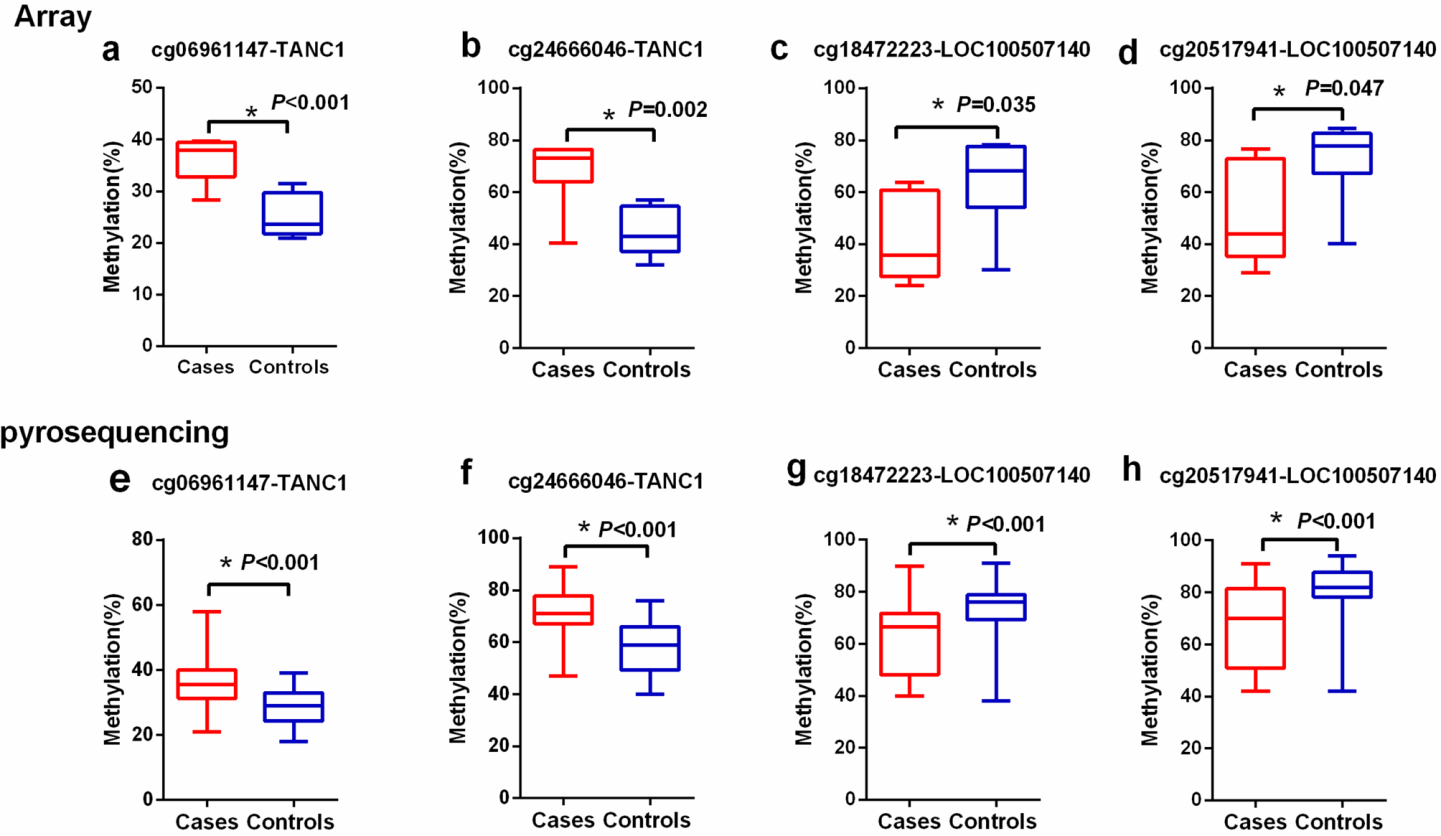
DNA methylation values of four CpGs sites measured by the EPIC BeadChip (top) and by bisulfite pyrosequencing (bottom). DNA methylation values of cg06961147 **(a)**, cg24666046 **(b)**, cg18472223 **(c)**, and cg20517941 **(d)** were measured using EPIC BeadChip. DNA methylation values at each CpG site by percentage were measured by pyrosequencing of the ADLI patients (red) and controls (blue) in cg06961147 **(e)**, cg24666046) **(f)**, cg18472223 **(g)**, and cg20517941 **(h).**

**Figure 6:**
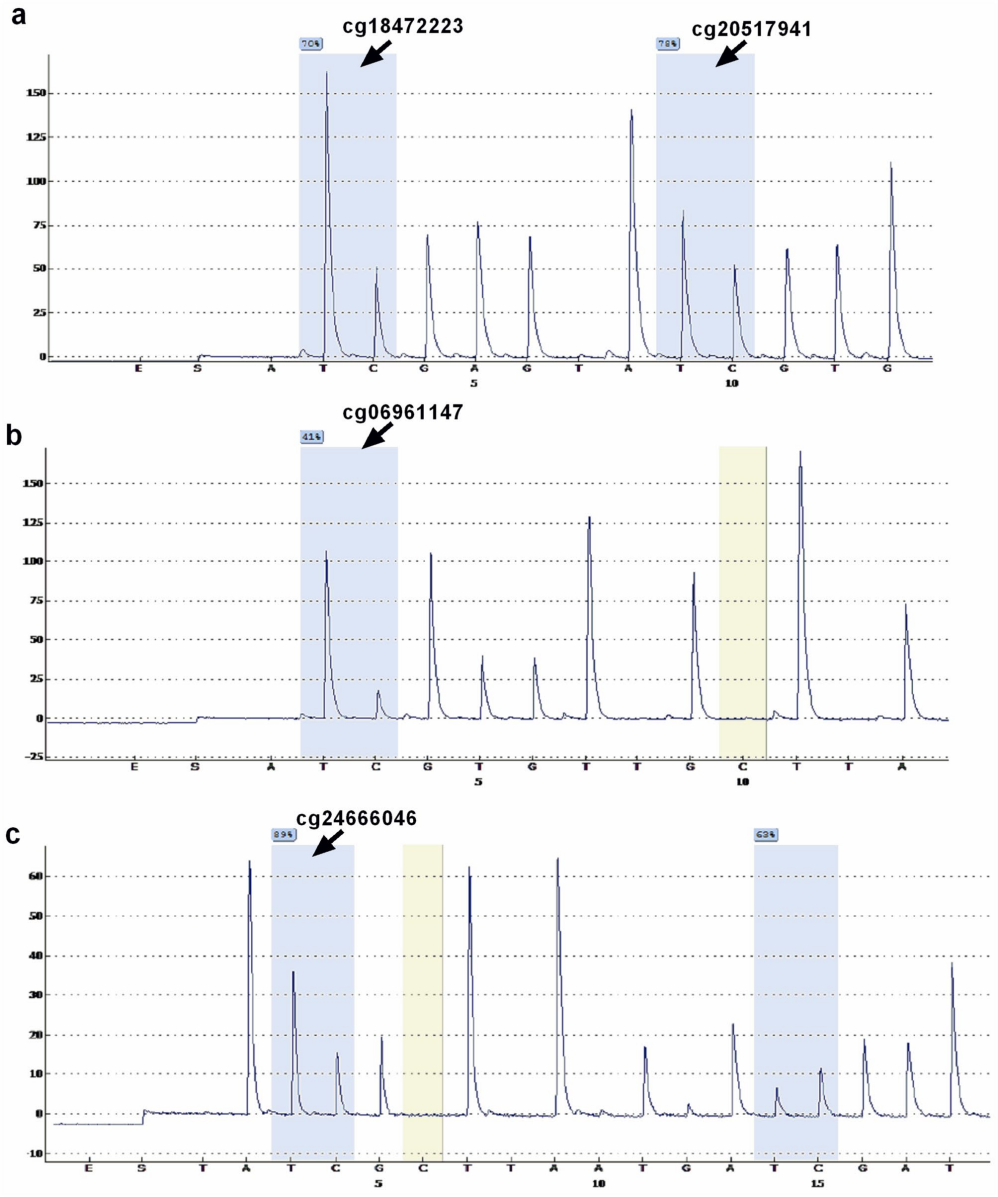
Representative images of pyrosequencing for four differentially methylated loci in one patient. Images of pyrosequencing were generated for cg18472223 and cg20517941 **(a)**, cg06961147 **(b)**, and cg24666046 **(c).**

The pyrosequencing results of two CpG sites (cg18472223 and cg20517941) within the promoter region of LOC100507140 and two CpG sites (cg18472223 and cg20517941) within the promoter region of TANC1 displayed lower and higher methylation levels in ADLI samples, respectively, consistent with the EPIC BeadChip array (Fig. 5). Representative images of pyrosequencing for four differentially methylated loci in one patient are shown in Fig. 6.

### Correlation analysis results of various indicators

The mRNA expression levels of TANC1 gene was negatively correlated with ALT and AST (*ρ* = − 0.818 and − 0.800, respectively; all *P* < 0.001), while the mRNA expression levels of LOC100507140 was positively correlated with ALT and AST (*ρ* = − 0.893 and − 0.824, respectively; all *P* < 0.001). The mRNA expression levels of TANC1 gene was negatively correlated with the methylation levels of the two CpG sites (cg06961147 and cg24666046), and the correlation coefficient *ρ* was − 0.500 and − 0.515, respectively (all *P* < 0.001). Similarly, the mRNA expression levels of LOC100507140 gene was negatively correlated with the methylation levels of the two CpG sites (cg18472223 and cg24666046), and the correlation coefficient *ρ* was − 0.383 and − 0.368, respectively (all *P* < 0.001).

### Diagnostic value of ADLI-associated differentially methylated sites

**Figure 7:**
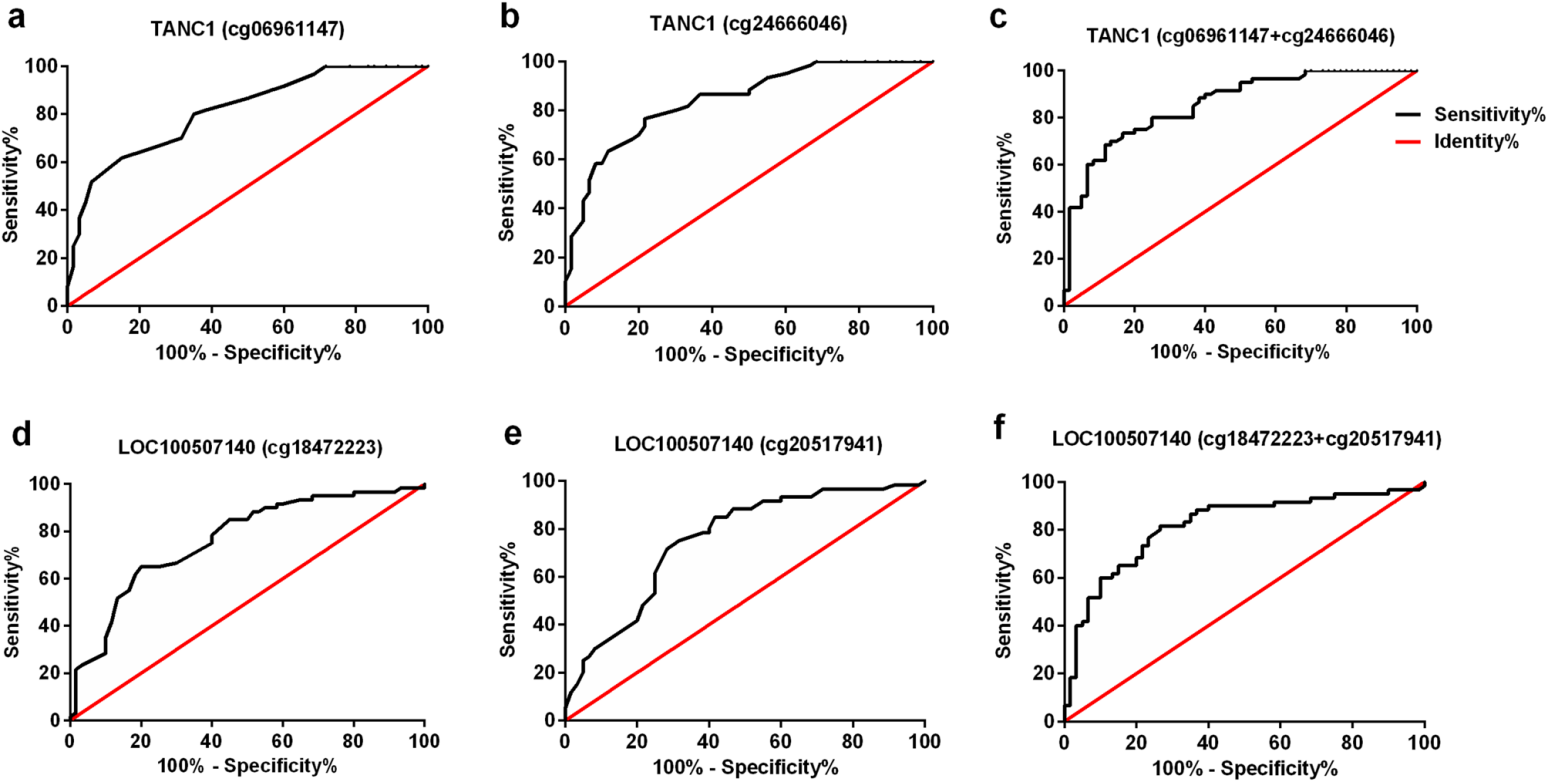
The receiver operating characteristic (ROC) curve for the discrimination of ADLI and non-ADLI. ROC curves were generated for cg06961147 **(a)**, cg24666046 **(b)**, and in combination **(c)** of the TANC1 gene. ROC curves of the cg18472223 **(d)**, cg20517941 **(e)**, and combination **(f)** of the LOC100507140 gene were also generated.

The diagnostic values of TANC1 (cg06961147, cg24666046, and in combination) and LOC100507140 (cg18472223, cg20517941, and in combination) were identified using ROC curves. As shown in Table 3 and Fig. 7, the area under the curve (AUC) of TANC1 (cg06961147, cg24666046, and in combination) were 0.812 (95% CI 0.737–0.887), 0.842 (95% CI 0.774–0.911), and 0.857 (95% CI 0.792–0.923), respectively. The AUCs of LOC100507140 (cg18472223, cg20517941, and in combination) were 0.765 (95% CI 0.680–0.851), 0.754 (95% CI 0.667–0.842), and 0.818 (95% CI 0.739–0.896), respectively. These results indicated that the combination of the two loci of TANC1 genes, cg06961147 and cg24666046, could be used to better distinguish between the ADLI and non-ADLI groups.

**Table 3:** The ROC curve parameters of four differentially methylated locus and their combinations for discriminating between ADLI and non-ADLI.

	AUC (95% CI)	Youden index	cut-off point	Sensitivity (%)	Specificity (%)
cg06961147	0.812 (0.737–0.887)	0.467	30.50	85.00	61.67
cg24666046	0.842 (0.774–0.911)	0.517	66.50	78.33	76.67
cg06961147 + cg24666046	0.857 (0.792–0.923)	0.567	0.44	86.67	70.00
cg18472223	0.765 (0.680–0.851)	0.450	72.50	80.00	65.00
cg20517941	0.754 (0.667–0.842)	0.433	79.50	71.67	71.67
cg18472223 + cg20517941	0.818 (0.739–0.896)	0.550	0.470	73.33	81.67

### Ingenuity pathway analysis of the TANC1 gene

**Figure 8:**
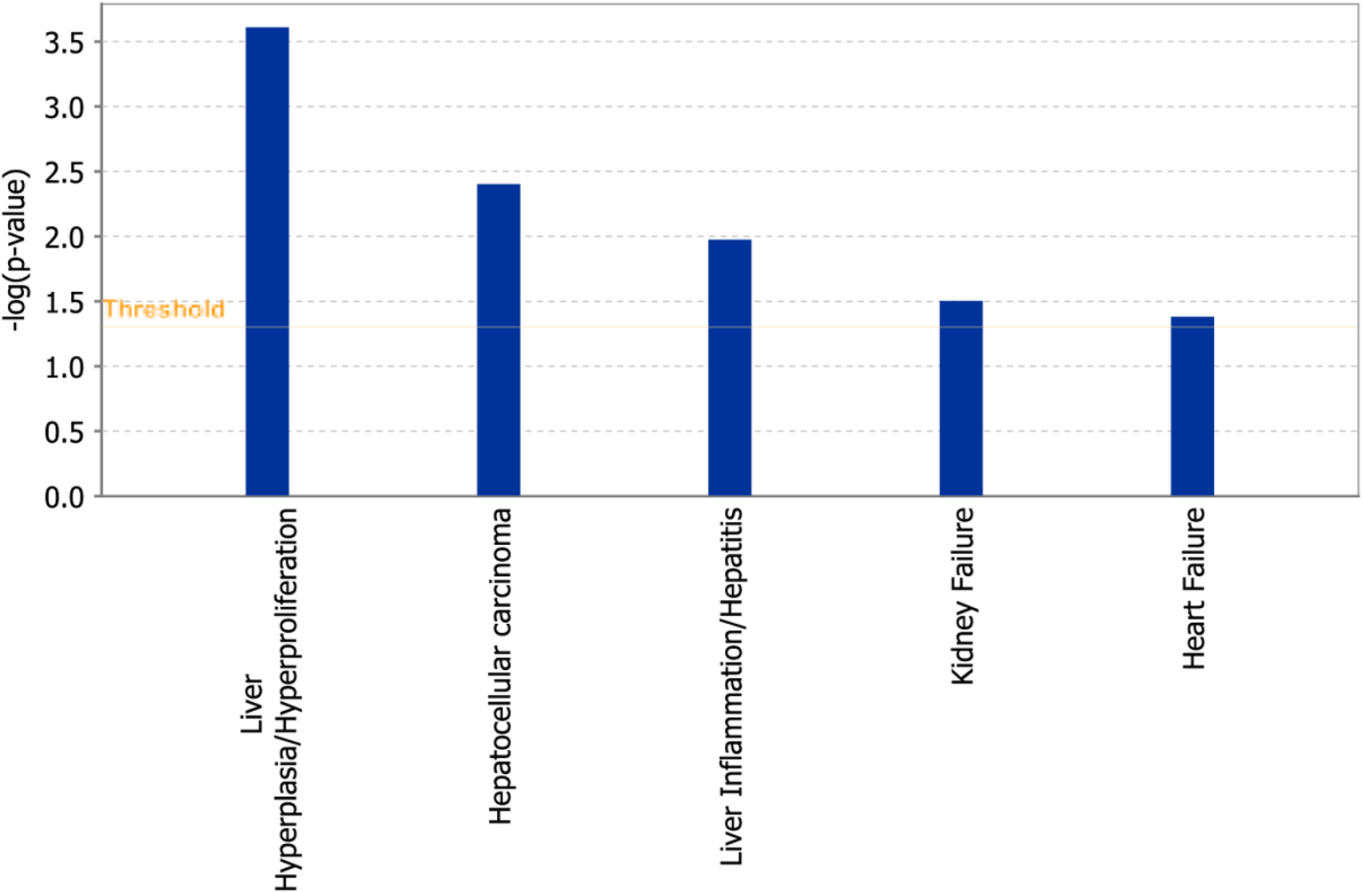
Significant canonical pathways identified by Ingenuity Pathway Analysis (IPA). Genes related to TANC1 gene (Supplementary Fig. S1) were significantly enriched in the above 5 pathways. The image was generated through the use of IPA (QIAGEN Inc. software version 65367011, https://www.qiagenbioinformatics.com/products/ingenuity-pathway-analysis)^23^.

A total of 77 upstream and downstream genes related to the TANC1 gene were enriched in IPA (see Supporting Information Fig. S1). These enriched genes were selected for the analysis of canonical pathways using IPA. The IPA analysis results in the identification of five significant canonical pathways that were significantly enriched with these genes (Fig. 8). The pathways included liver hyperplasia/hyperproliferation, hepatocellular carcinoma, liver inflammation/hepatitis, kidney failure, and heart failure. The results showed that these genes were mainly enriched significantly in several pathways related to liver metabolism, suggesting that TANC1 is related to liver disease.

## Discussion

In this study, the genome-wide patterns of DNA methylation in the blood of ADLI patients and non-ADLI controls were analyzed. Our results suggest that DNA methylation differs significantly between these two groups. Furthermore, using pyrosequencing in a larger sample size, we demonstrated that the expression and methylation of LOC100507140 and TANC1 could be used to distinguish between ADLI patients and the controls. These findings indicate that changes in DNA methylation levels may be related to alterations in the expression of certain genes during ADLI occurrence. These findings also suggest that the abnormal expression of DNA methylation could be used as an indicator of ADLI, similar to the predictive effect of abnormal DNA methylation in other diseases^24–26^.

To our knowledge, this study is the first to identify distinct differential DNA methylation patterns in blood samples from patients with ADLI and non-liver injury using the EPIC BeadChip array platform. The EPIC BeadChip array has the advantage of covering the whole genome, including the gene promoter region, the gene coding region, CpG islands, and the enhancer regions found in the ENCODE^27^ and FANTOM5^28^ project. In our results, fundamental differences in DNA methylation patterns between the ADLI and non-ADLI patients were elucidated by PCA (Fig. 1b). Further analysis complemented these results by identifying site-specific CpG percentage (%) methylation changes that were responsible for pattern differentiation between the two groups (Fig. 2). The volcano plot in Fig. 2a shows the full distribution of the observed differential sites. The statistically significant CpG sites (N = 806) in the methylation heatmap (Fig. 2b) show a distinct pattern between the groups. The combined results provide a more comprehensive display of the genome-wide methylation of ADLI. Our understanding these epigenetic changes will enable the use of epigenetic biomarkers for diagnosis of disease in early stages. In general, our results display the genome-wide methylation of ADLI more comprehensively.

The genomic distribution of the dmCpGs suggests that the hypermethylation of dmCpGs may vary depending on the location: based on dmCpGs in CpG island-related or gene location-related status, dmCpGs were mainly observed in the open sea of the CpG-poor regions and the gene bodies, respectively. This phenomenon underscores the value of screening technologies that accurately examine CpG-sparse regions. In addition, the fact that the majority of methylation changes were identified in gene bodies emphasized that we cannot focus solely on methylation sites located in CpG-denser regions, such as CpG islands and gene promoters. Evidence is increasingly suggesting that DNA methylation in gene bodies is able to promote oncogene expression^29,30^, such that gene body methylation may serve as a therapeutic target for the treatment of cancer^31,32^. Our results suggest that the DNA methylation of gene bodies will be an important topic for future studies in the field of cancer research.

Notably, we found that the combination of two CpG sites (cg06961147 and cg24666046) of the TANC1 gene could act as potential biomarkers for the diagnosis of ADLI cases and non-ADLI controls. This is a novel discovery. Although there are currently very few reports on TANC1 research, many studies have reported that TANC1 is an important synaptic scaffold protein that plays a critical role in regulating the density of synaptic spines and excitatory synapse strength^33^. Studies have also indicated that TANC1 is a candidate gene for neurodevelopmental disorders (NDD)^34,35^. Furthermore, the TANC1 locus can influence the development of late radiation-induced damage^36^. In the present study, the TANC1 gene was analyzed with IPA to identify any associated significant pathways, upstream regulators, diseases, and functions. A total of 77 upstream and downstream genes related to the TANC1 gene were found to be enriched in IPA (Fig. 8a). Our results indicated that these genes were significantly enriched in several pathways related to liver metabolism, including liver hyperplasia/hyperproliferation, hepatocellular carcinoma, and liver inflammation/hepatitis (Fig. 8b), suggesting that the TANC1 gene is related to liver disease. To date, the biochemical properties of TANC1 proteins remain largely unknown, and there is currently no research on the relationship between TANC1 methylation and diseases. Since methylated DNA is very stable^37^ and can be detected in clinical blood samples^38,39^, it is a promising target for use in disease diagnostics. Taken together, our results indicate that DNA methylation is a promising diagnostic target for ADLI.

Our study contains a number of limitations. First, our sample size was relatively small, with limited power; hence, future studies should investigate whether the CpG sites identified in this study can be replicated in an independent population. In addition, given the large number of dmCpGs found and distributed at different locations in the present study, we were unable to determine all of the dmCpGs. Therefore, we only performed mRNA and pyrosequencing verification of dmCpGs in the TSS1500 region. The dmCpGs located in other regulatory regions will need to be validated in future studies. Finally, although we identified two differentially methylated genes, LOC100507140 and TANC1, we did not study their possible mechanisms of action further. Despite these limitations, this study is the first to use the EPIC BeadChip array platform identify the DNA methylation signatures associated with ADLI.

In summary, the distinctive differences in DNA methylation patterns between ADLI and non-ADLI patients were the main finding of our study. The expression of LOC100507140 and TANC1, as the differentially methylated genes, was found to vary significantly during the occurrence of ADLI. More importantly, we found that the combination of the hypermethylated differentially methylated site cg06961147 and cg24666046 in TANC1 provides a potential target for the diagnosis of ADLI.

## Supplementary Information

Supplementary Information.

## Data Availability

The datasets generated during and/or analyzed during the current study are available from the corresponding author on reasonable request.

## Abbreviations

ADLI: Anti-tuberculosis drug-induced liver injury
TB: Tuberculosis
DOTS: Directly observed treatment short-course chemotherapy
H: Isoniazid
R: Rifampicin
Z: Pyrazinamide
E: Ethambutol
EPIC: MethylationEPIC BeadChip
dmCpGs: Differentially methylated CpG sites
ATS: American thoracic society
ULN: Upper limit of normal
BMI: Body mass index
ALT: Alanine aminotransferase
AST: Aspartate aminotransferase
ALP: Aspartate aminotransferase
TBIL: Total bilirubin
ALB: Albumin
TP: Total protein
SD: Standard deviation
IQR: Median with interquartile range
qRT-PCR: Quantitative reverse transcriptase polymerase chain reaction
PCA: Principal component analysis
TSS: Transcriptional start site
UTR: Untranslated region
CpG: Cytosine-guanine dinucleotide
IPA: Ingenuity Pathway Analysis
