# Supplementary material for: TANC1 methylation as a novel biomarker for the diagnosis of patients with anti-tuberculosis drug-induced liver injury
Source: Sci Rep. 2021 Aug 31;11:17423. doi: 10.1038/s41598-021-96869-5 (PMC8408132; doi:10.1038/s41598-021-96869-5)
Supplement: Supplementary file 1 — Supplementary Information. [file 41598_2021_96869_MOESM1_ESM.docx]

TANC1 methylation as a novel biomarker for the diagnosis of patients with anti-tuberculosis drug-induced liver injury

Dongxue Wu, Yuhong Li, Qi Ren, Shengfei Pei, Lin Wang, Luming Yang, Yingzhi Chong, Shufeng Sun, Jinqi Hao, Fumin Feng

**Supporting Information Table S1** Primer sequences for Real-time PCR

| Gene | Primer sequence (5’-3’) | | Annealing temperature (℃) |
| --- | --- | --- | --- |
| LOC100507140 | | Sense: TTTTTGCTTCTAATGAGACCTGACTTT | 60 |
|  | | Antisense: AGGAGAAAAACAGACCAACAGAACA |  |
| TANC1 | | Sense: CCTCTGATGTCATTCTTCAACGTT | 60 |
|  | | Antisense: AGCAAAACTCATGGAAAGCCTATAA |  |
| C1orf141 | | Sense: GATTCAGGACCAAGAAAGGCATAGT | 60 |
|  | | Antisense: AGAGTATTGATGTTATTTGGCTTCCA |  |
| C22orf39 | | Sense: CCAGCAGGCTTCACTTCTTAGG | 60 |
|  | | Antisense: TGTTCTGTAGCTGTGTGCATTTCTT |  |
| CD177 | | Sense: TCGCCCCATCTGAGCAA | 60 |
|  | | Sense: GTCCCTGTCGTTAAGCAATGC |  |
| FMOD | | Antisense: TGGAGGAGAGTTAGCCCAAGTATAG | 60 |
|  | | Sense: AAGCATACACAGTATCAGGGATGTGA |  |
| HCG27 | | Antisense: CGTGCATTTCCACCTGTTCTC | 60 |
|  | | Sense: CACCCAGGGCTGTGTTTTTT |  |
| LOC102723376 | | Sense: CGGCTCCCTCGTACACTCTCT | 60 |
|  | | Antisense: CTTCTCTCCTCTTTGGCTCACATAG |  |
| PKD1L2 | | Sense: ACAAGCTTTGCCCAAGGACTAA | 60 |
|  | | Antisense: CGTCCCCACTGGTAGATGGT |  |
| BIRC7 | | Sense: TGCCCCTCTCTGCCTGTTC | 60 |
|  | | Antisense: GACACCAGCTCTGCCATCCT |  |
| GAPDH | | Sense:GAAGGTCGGAGTCAACGGATT | 60 |
|  | | Antisense: CCTGGAAGATGGTGATGGGAT |  |

**Supporting Information Table S2** Primer sequence and polymerase chain reaction (PCR) conditions used for each pyrosequencing assay

|  | cg18472223, cg20517941 (LOC100507140) | cg06961147 (TANC1) | cg24666046 (TANC1) |
| --- | --- | --- | --- |
| Primer sequences(5′-3′) |  |  |  |
| Forward | AGTGTTTTTTATTTATTTGGGATTTAGGA | AGAGTAAAAATTAGGATAGGGATTAG | TTGGTGTGTTTGGAGTAGAGAAT |
| Reverse-Bio | AAACAAACAATACCCCTTCCTTTAAAACTC | CACAAAACAACCACAATTCTAATTAATCCT | ATAAACACCCCTCCTACTAATATC |
| Sequence | ATTTGGGATTTAGGAAAAG | GGATAGGGATTAGGGA | GTAATTTTTAATTTTGTTGTGGT |
|  |  |  |  |
| PCR cycling conditions |  |  |  |
| Step 1(De-naturation) | 98 °C /10 sec | 98 °C /10 sec | 98 °C /10 sec |
| Step 2 (Annealing) | 55 °C /30 sec | 56 °C /30 sec | 58 °C /30 sec |
| Step 3(Extension) | 72 °C /30 sec | 72 °C /30 sec | 72 °C /30 sec |
| Step 4 | Go to 1, 39 times | Go to 1,39 times | Go to 1, 39 times |
| Step 5(Extension) | 72 °C /1 min | 72 °C /1 min | 72 °C /1 min |

**Supporting Information Table S3** Genes containing multiple differentially methylated CpGs in ADLI

| Ucsc Refegene | No.of CpGs | Ucsc Refegene | No.of CpGs | Ucsc Refegene | No.of CpGs |
| --- | --- | --- | --- | --- | --- |
| ADAP1 | 3 | FAM19A2 | 2 | PCDHB3 | 2 |
| AIM2 | 2 | FMOD | 10 | PKD1L2 | 4 |
| AKAP13 | 2 | HCG27 | 2 | PQLC1 | 2 |
| BIRC7 | 3 | HABP2 | 2 | PRKAR1B | 2 |
| BRSK2 | 3 | HDAC4 | 2 | PTPRN2 | 4 |
| C1orf141 | 2 | HLA-B | 2 | RAD51B | 2 |
| C21orf91-OT1 | 2 | HLA-C | 7 | RAP1GAP2 | 3 |
| C22orf39 | 2 | HLA-DQB1 | 3 | RBM19 | 2 |
| CACNB2 | 2 | HOOK2 | 4 | SGMS1 | 3 |
| CASC15 | 2 | IDI1 | 2 | SLMO2 | 2 |
| CD177 | 2 | KCNQ5 | 2 | TANC1 | 3 |
| CDH13 | 4 | LOC100507140 | 2 | TEX14 | 2 |
| CHL1 | 2 | LOC102723376 | 2 | TG | 2 |
| CNTNAP4 | 2 | MICA | 2 | TK1 | 2 |
| CROCC | 2 | MOBP | 4 | TMCO3 | 4 |
| DDR1 | 3 | MYT1L | 3 | TNFAIP8 | 3 |
| DPCR1 | 2 | OXNAD1 | 2 | VSTM1 | 3 |
| FAM125B | 2 | PCBP1-AS1 | 2 |  |  |


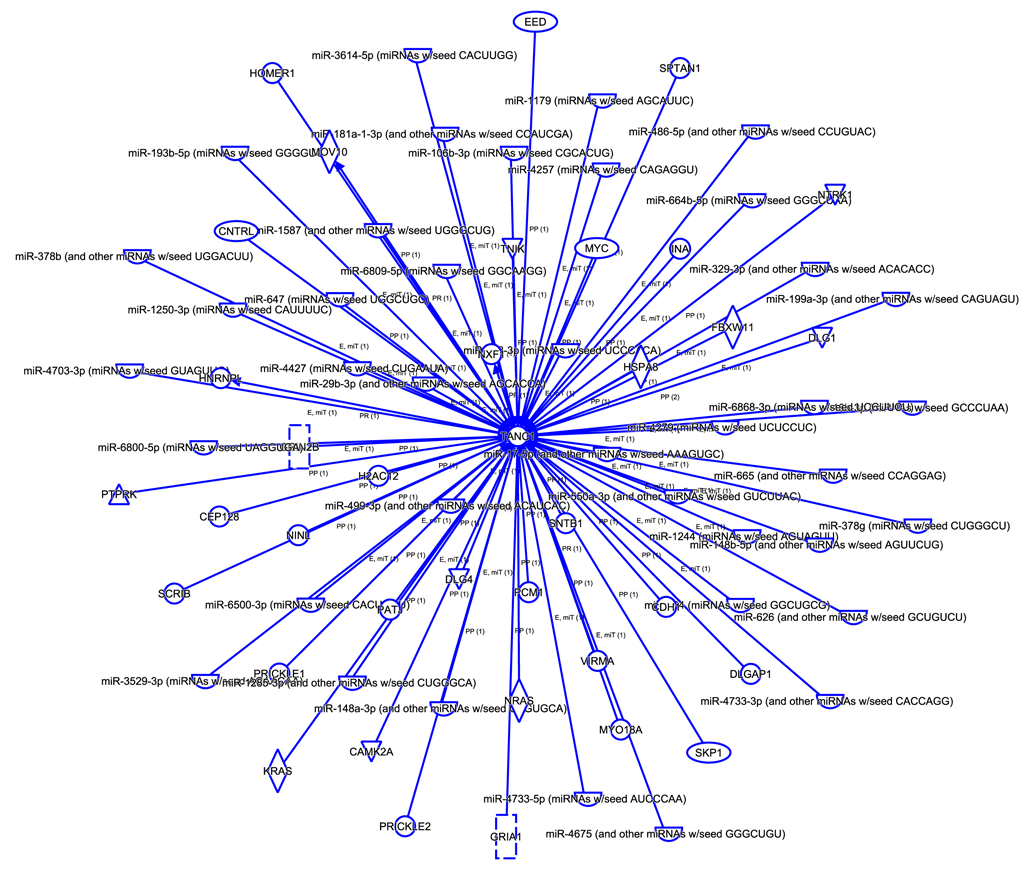


**Supporting Information Fig. S1** Upstream and downstream genes of TANC1 enriched by Ingenuity Pathway Analysis (IPA). The network was generated through the use of IPA (QIAGEN Inc. software version 65367011, https://www.qiagenbioinformatics.com/products/ingenuity-pathway-analysis) ^[23]^.
